# Current News and Opinion

**Published:** 1884-12

**Authors:** 


### LEGAL DECISIONS.

A dentist in San Francisco, who forcibly removed some fillings the payment for which was in dispute, was condemned by the courts to pay damages in the sum of $217.50. It has also been decided that a dentist cannot retain an unpaid-for set of teeth that may come into his hands again for alterations or repairs.

described by Dr. Allport, though in that case the teeth were not extracted, but the plate was made to fit over them.

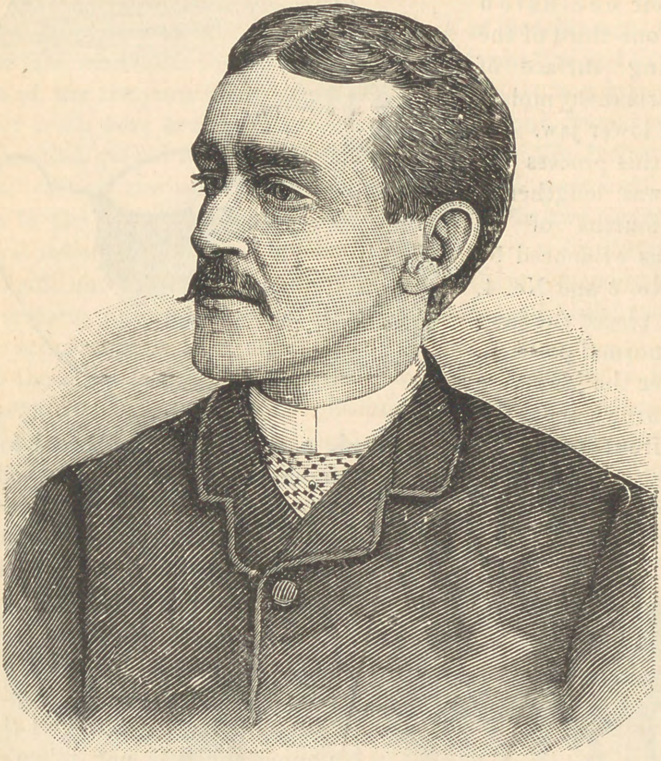

Cut No. 4.

*Dr. Williams*—Moved that, owing to the lateness of the hour and the desire of many to leave by the early evening trains, discussion of the paper be passed.

The section then adjourned.

---

### PROGRESS UNDER DIFFICULTIES.

A dental journal, to be called the *Dental Review,* is to be established in Russia. What can be its scientific status in a country in which, by an imperial decree, the works of Agassiz, Huxley, Lubbock, Herbert Spencer, Vogt, Zimmerman, and Charles Darwin, are under interdict; a land in which a demonstrated scientific fact is not truth unless it receives the approval of the church through its head?

### Current News and Opinion.

### DENTAL SCHOOL IN BERLIN.

A new dental school, called "The Dental Institute of the Royal University," has been opened in Germany, by direction of the *Kultus Ministerium,* with Professor Dr. Busch as director, and Professors Dr. W. D. Miller, and Dr. J. Paetsch, as Professors of Operative and Clinical Dentistry. The mechanical department will be in charge of Zahnarzt C. Sauer.

It should be remembered that in Germany the title of Professor is conferred by government. The "Kultus Ministerium" is a department of government, and under the charge of a minister. There is a Minister of State, a Minister of War, etc., and the "Kultus" Minister has charge of all educational and church matters, art, and everything pertaining to culture. A "Professor," then, in Germany, is an officer under the government. We heartily congratulate our *confrere* upon this deserved and distinguished honor, and in this connection present the following appreciative letter from one of his colleagues:

BERLIN, October 19, 1884.

*Editor Independent Practitioner:*

One of your countrymen and collaborators of the INDEPENDENT PRACTITIONER, Dr. W. D. Miller, who has been a practicing dentist in Berlin for a few years past, has now received from our "Kultus Ministerium" the honorable distinction of "Royal Professor in the Berlin University."

Dr. Miller is the first foreigner on whom such an honor has been bestowed, and he owes this distinction chiefly to his scientific investigations, and to his valuable essays, the most of which were first published in your journal. I send you this that you may know that we in Germany recognize the attainments and merit of your countryman.

Very truly yours,

J. PAETSCH, M. D., D. D. S.,

*Balt. Dent. Coll.*, 1865.

---

### OBTUSE.

*Editor Independent Practitioner:*

In the November number of *Items of Interest* "J. R. Trust" gives evident indications that he can neither see nor take a joke. He must be one of the Scotchmen of whom Dr. Johnson tells, who required a surgical operation to get a jest into their heads. Hereafter I *trust* that you will imitate the perspicacity of the lamented Artemus Ward, and to assist his comprehension add at the bottom of your humorous articles "This is a Goak"—"This, and nothing more."

"ONE WHO HAS BEEN THERE."

---

### MURIATE OF COCOAINE.

Too late for insertion in this number, we have received from Dr. Julien W. Russell a report upon the use in dental practice of this newly discovered local anæsthetic. He finds it of great utility in all cases of surgery in the mouth, for obtunding sensitive dentine, for rendering insensible the tissues of the mouth in taking impressions for patients who are liable to nausea, and especially for the painless removal of living pulps. It is of little benefit in the extraction of teeth. We regret that his article did not come in time for insertion entire.

---

### THE OHIO STATE DENTAL SOCIETY.

This association was reorganized and chartered Oct. 31,1884. It will be officered as follows:

President, C. H. James, of Cincinnati; Vice-President, H. H. Harrison, of Cadiz; Secretary, J. R. Callahan, of Hillsboro; Treasurer, G. W. Keely, of Oxford. It will meet on the last Wednesday of October, 1884, at Chillicothe, Ohio.

J. R. CALLAHAN, *Sec.*

(31.) Will some one give a cure for mouth-breathing ? I have a case that I am very desirous to cure, and am at my wits' end as to the proper course to pursue. C. L. H., Kansas.

(32.) Will some dentist of experience briefly describe the method he has found most successful in capping exposed pulps, and so benefit many younger members of the profession ? A. B. M.

(33.) The effect of nicotine upon the mucous membrane of the mouth is fairly well known. Will some practitioner who has experimented (if there be such), state the effects of nicotine upon the normal tooth, or upon the dentine when through caries it has been denuded of enamel ? C. E. H., P.

(34.) I have a case that puzzles me. A boy, hardly eight years of age, received a blow on a left superior central incisor, which has become somewhat discolored. Is it safe to open into the pulp cavity and remove the pulp in so young a tooth ? Iowa.

(35.) It has been stated that dentine is less sensitive to the touch of an excavator where the cavity is kept dry than when prepared under moisture. Will some reader of your excellent journal please explain why this is so ?

B. L. S.

(36.) Have our scientists settled the question as to whether carbolic acid is an *antiseptic* or *disinfectant?* or is it neither ? W. S. R.

### Answers.

Trouble (26.) In taking your bite your upper plate springs from the roof of the mouth. You replace it on the cast, and press it down to fit. This will cause the molar teeth, when it is finished, to be too long every time. If the plate springs from the roof of the mouth when the bite is taken, leave it so on the cast, and you will have no *trouble.* G. M. Merritt, Jersey City.

D. D. S. (27.) There is nothing better, in my opinion, for coating an impression than a not too thick solution of red shellac, the impression to be dipped in water before pouring. You are thus enabled to see just where the line of division is, or to carefully cut the impression away from the cast where there are undercuts, and from between teeth. If the shellac coat be allowed to dry on the impression, it will not peel off or separate.

Another D. D. S.

L. (29.) When the blood flows from the socket of an extracted tooth, little is needed save to roll up some cotton lint into a hard plug, a little larger than the socket and of a shape to fit, and force it down to place, and hold it there until a clot is formed in the vein or artery. Do not use any of the preparations of iron as hemostatics, but, if necessary, place a cork, properly cut, upon the cotton to hold it firmly in place, and bandage the jaws together. The dangerous cases are those in which there is a hemorrhagic diathesis, and then the blood does not come from the socket, but oozes from the gums anywhere.

Western Dentist.

